# Supplementary material for: Identification of photoperiod-induced specific miRNAs in the adrenal glands of Sunite sheep (Ovis aries)
Source: Front Vet Sci. 2022 Jul 22;9:888207. doi: 10.3389/fvets.2022.888207 (PMC9354845; doi:10.3389/fvets.2022.888207)
Supplement: Supplementary file 1 [file Data_Sheet_1.ZIP › Supplementary file/miRDeep2 file.zip!/miRDeep2 file/Know_Structure/SPLP42A1/oar-mir-493.pdf]

miRBase precursor : oar-mir-493
Total read count : 14519
oar-miR-493-5p read count : 10352
oar-miR-493-3p read count : 4167
remaining reads : 0

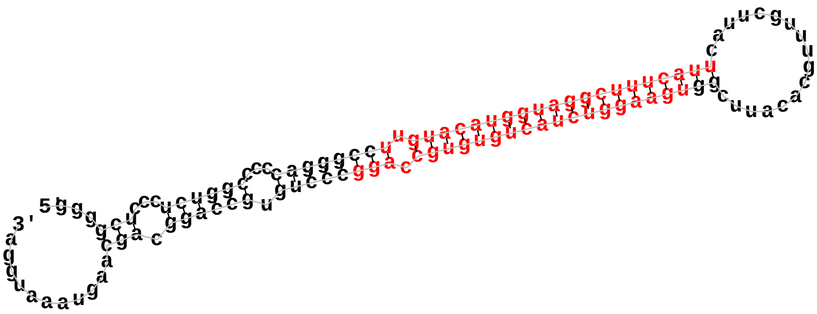

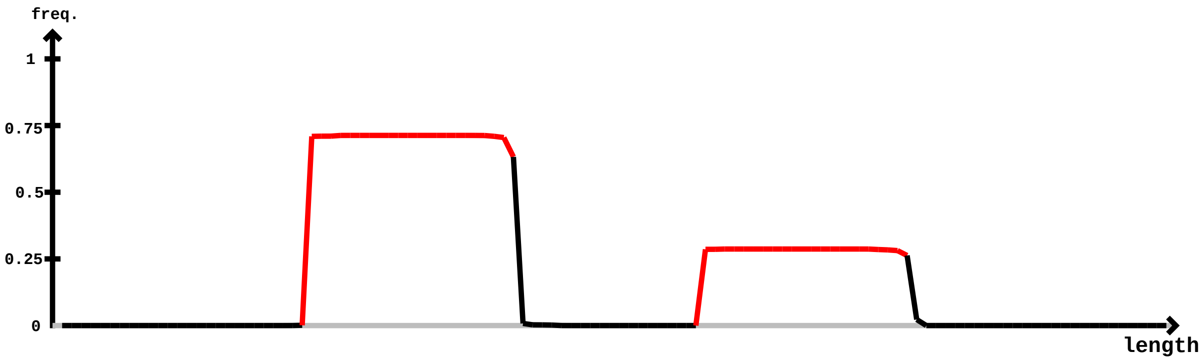

oar-miR-493-5p

oar-miR-493-3p

5'- ggggcucccucuggcccccagggccuuguacaugguaggcuuucauucauucguuugcacauucggugaaggucuacugugugccaggcccugugccaggcagcaaguaaaugga -3' exp

...(((...((((((...((((((((.((((((((((((((((((((.................)))))))))))))))))))).)))))))).)))))).)))...........	reads	mm	sample
.......................ccuuguacaugguaggcuuuc...	1	0	seq
.......................ccuuguacaugguaggcuuuca..	1	0	seq
........................cuuguacaugguaggcuuucau...	2	0	seq
........................cuuguacaugguaggcuuucauu..	12	0	seq
.........................uuguacaugguaggcuu...	2	0	seq
.........................uuguacaugguaggcuuu..	4	0	seq
.........................uuguacaugguaggcuuuc...	43	0	seq
.........................uuguacaugguaggcuuuca..	65	0	seq
.........................uuguacaugguaggcuuucau...	1052	0	seq
.........................uuguacaugguaggcuuucauu..	9044	0	seq
.........................uuguacaugguaggcuuucauuc...	73	0	seq
.........................uuguacaugguaggcuuucauuca..	1	0	seq
..........................uguacaugguaggcuuucauu..	8	0	seq
..........................uguacaugguaggcuuucauuc...	2	0	seq
..........................uguacaugguaggcuuucauuca..	1	0	seq
...........................guacaugguaggcuuucauu..	2	0	seq
............................uacaugguaggcuuucauuc...	1	0	seq
............................uacaugguaggcuuucauuca..	2	0	seq
............................uacaugguaggcuuucauucau...	9	0	seq
............................uacaugguaggcuuucauucauu..	24	0	seq
............................uacaugguaggcuuucauucauuc...	1	0	seq
.............................acaugguaggcuuucauucauu..	2	0	seq
....ugaaggucuacugugug................................	1	0	seq
....ugaaggucuacugugugc...............................	15	0	seq
....ugaaggucuacugugugU...............................	12	1	seq
....ugaaggucuacugugugcc..............................	19	0	seq
....ugaaggGcuacugugugcc..............................	1	1	seq
....ugaaggucuacugugugcca.............................	38	0	seq
....ugaaUgucuacugugugccag............................	1	1	seq
....ugaaggCcuacugugugccag............................	1	1	seq
....ugaaggucuacuAugugccag............................	1	1	seq
....ugaaggucuacugugugccag............................	258	0	seq
....ugaaggucuGcugugugccag............................	3	1	seq
....ugaagAucuacugugugccag............................	1	1	seq

```
                         oar-miR-493-5p
                                                                  oar-miR-493-3p
ggggcucccucuggcccccagggccuuguacaugguaggcuuucauucauucguuugcacauucggugaaggucuacugugugccaggcccugugccaggcagcaaguaaaugga

..................................................................ugaaUgucuacugugugccagg...........................  2     1     seq
..................................................................ugaaggucuacuAugugccagg...........................  1     1     seq
..................................................................ugaaggucuacugugugccagg...........................  3439  0     seq
..................................................................ugaaggCcuacugugugccagg...........................  12    1     seq
..................................................................ugaagguUuacugugugccagg...........................  2     1     seq
..................................................................ugaaggucuGcugugugccagg...........................  14    1     seq
..................................................................ugaaggucuacuguguUccagg...........................  1     1     seq
..................................................................ugaagAucuacugugugccagg...........................  6     1     seq
..................................................................ugaagguUuacugugugccaggc..........................  2     1     seq
..................................................................ugaaggucuacugugugccaggc..........................  313   0     seq
..................................................................ugaaggucuGcugugugccaggc..........................  4     1     seq
..................................................................ugaaggCcuacugugugccaggc..........................  4     1     seq
...................................................................gaaggucuacugugugccagg...........................  1     0     seq
....................................................................aaggucuacugugugccag............................  1     0     seq
....................................................................aaggucuacugugugccagg...........................  8     0     seq
....................................................................aaggucuacugugugccaggc..........................  6     0     seq
```
